# Computer-Aided Directed Evolution Generates Novel AAV Variants with High Transduction Efficiency

**DOI:** 10.3390/v15040848

**Published:** 2023-03-26

**Authors:** Zengpeng Han, Nengsong Luo, Fei Wang, Yuxiang Cai, Xin Yang, Weiwei Feng, Zhenxiang Zhu, Jie Wang, Yang Wu, Chaohui Ye, Kunzhang Lin, Fuqiang Xu

**Affiliations:** 1 State Key Laboratory of Magnetic Resonance and Atomic and Molecular Physics, Key Laboratory of Magnetic Resonance in Biological Systems, Wuhan Center for Magnetic Resonance, Innovation Academy for Precision Measurement Science and Technology, Chinese Academy of Sciences, Wuhan 430071, China; 2 University of Chinese Academy of Sciences, Beijing 100049, China; 3 Shenzhen Key Laboratory of Viral Vectors for Biomedicine, Key Laboratory of Quality Control Technology for Virus-Based Therapeutics, Guangdong Provincial Medical Products Administration, NMPA Key Laboratory for Research and Evaluation of Viral Vector Technology in Cell and Gene Therapy Medicinal Products, the Brain Cognition and Brain Disease Institute (BCBDI), Shenzhen Institute of Advanced Technology, Chinese Academy of Sciences, Shenzhen-Hong Kong Institute of Brain Science-Shenzhen Fundamental Research Institutions, Shenzhen 518055, China; 4 Wuhan National Laboratory for Optoelectronics, Huazhong University of Science and Technology, Wuhan 430074, China; 5 iDrugDance Technology Co., Ltd., Shenzhen 518055, China; 6 ZhunAo Biotechnology Co., Ltd., Wuhan 430056, China; 7 Center for Excellence in Brain Science and Intelligence Technology, Chinese Academy of Sciences, Shanghai 200031, China

**Keywords:** adeno-associated virus, directed evolution, computer-aided design, *Cap* gene library, transduction efficiency

## Abstract

Adeno-associated viruses (AAVs) have become safe and effective tools for therapeutic in vivo gene drug delivery. Among many AAV serotypes, AAV2 is the most well-characterized. Although many studies have been carried out on the engineering of the capsid VR-VIII region, few attempts have been made in the VR-IV region. Here, we targeted amino acid positions 442–469 of the VR-IV region and established an engineering paradigm of computer-aided directed evolution, based on training samples from previous datasets, to obtain a viral vector library with high diversity (~95,089). We further examined two variants selected from the library. The transduction efficiency of these two novel AAV variants, AAV2.A1 and AAV2.A2, in the central nervous system was 10–15 times higher than that of AAV2. This finding provides new vehicles for delivering gene drugs to the brain.

## 1. Introduction

Adeno-associated viruses (AAVs) have become safe and effective tools for the in vivo delivery of therapeutic gene drugs due to their relatively low immune responses and broad tissue tropism [1,2]. The authorization of five AAV-based gene therapy drugs, comprising Glybera (AAV1), Luxturna (AAV2), Zolgensma (AAV9), Upstaza (AAV2), and Roctavian (AAV5), clearly demonstrated the enormous potential of AAV vectors for human gene therapy [3]. However, with a few exceptions, the transduction efficiency and specificity of natural capsids remain issues that impede certain critical applications and limit therapeutic purposes [4,5]. As synthetic biology progresses, the AAV capsid can be engineered to overcome these challenges through directed evolution, which is rooted in the simulation of natural evolution, thus generating genetic variants with enhanced specificity and advantageous characteristics through the process of mutation and selection [4,6].

Among many AAV serotypes, AAV2 is the most well-characterized; its *rep* gene and inverted terminal repeat (ITR) sequences are commonly used in the preparation of other serotypes of recombinant AAV [7]. The AAV capsid protein determines most of the properties of the vector. Several methods have been developed for the construction of a diversified *Cap* gene mutation library, including global *Cap* gene libraries implemented by DNA shuffling [6,8] or random point mutations [9] and site-specific libraries implemented by peptide display [10,11,12] or computer-aided design. In site-specific library design, the capsid VR-VIII region, especially the position between N587 and R588 at the spike around the 3-fold axis, has been most commonly used as a mutation introduction site in AAV2 [10,12,13,14]. Since this region is highly variable and on the outermost side of the capsid, it is suitable for the functional display of inserted peptides to retarget AAV2 to poorly susceptible cells [12,15,16,17]. Apart from this site, attempts to construct libraries in other regions of the *Cap* gene are rarely reported.

To better inform AAV capsid engineering, Ogden et al. [7] systematically examined the effects of all single-codon substitutions, insertions, and deletions across all 735 positions in the AAV2 *Cap* gene. On the one hand, some low-conserved regions suitable for library construction were revealed (e.g., positions 442–469 and 561–588) by the AAV2 capsid fitness landscape [7], and on the other hand, this information provides a training set for machine learning-aided library design. Thus, they focused on positions 561–588 and used these data to design mutant libraries based on machine learning [7,18].

Here, we targeted another position, 442–469, and established an engineering paradigm of computer-aided directed evolution based on training samples from the previous two public datasets [7,18]; then, we obtained a viral vector library with high diversity. As proof of principle, we further screened mutants from the library with higher transduction efficiency than AAV2, providing new vehicles for gene drug delivery in the brain.

## 2. Materials and Methods

### 2.1. Computer-Aided Design of the AAV2 Mutant Library

**Figure 1:**
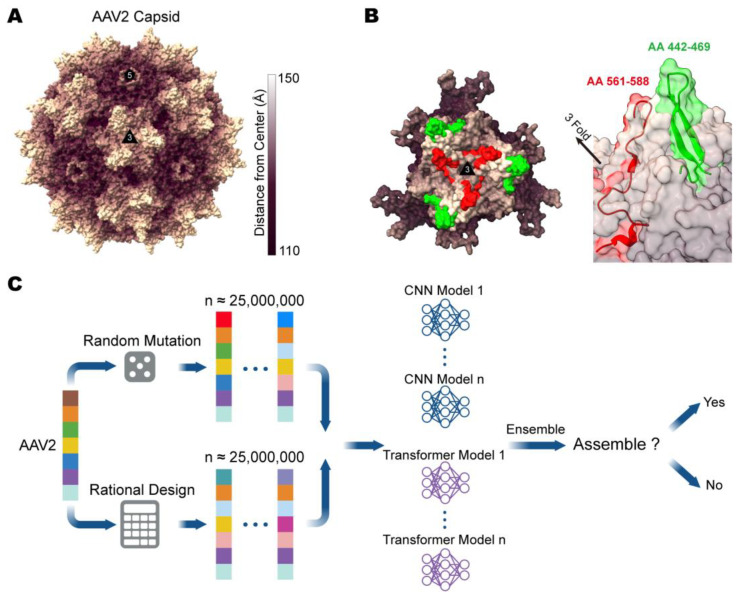
Capsid engineering locations and diagram of computer-aided design. (**A**) AAV2 capsid surface model demonstrates the location of the protruding loop structures in milky. The icosahedral 3- and 5-fold axes are indicated. (**B**) Left, an exterior capsid surface representation for AAV2 VP3 trimer. Right, an enlarged side view of the capsid shows the spike created by the AA442–AA469 (green) and the AA561–AA588 (red) variable regions at the icosahedral 3-fold axis. AA, amino acid. The figure (**A**,**B**) images were generated using UCSF ChimeraX v1.4 [20,21] (UCSF, San Francisco, CA, USA). (**C**) Schematic diagram of computer-aided design.

The most commonly modified position within the AAV2 capsid protein is the surface-exposed loop (Figure 1A), which contains amino acids (AAs) 561–588, because it is the site of heparan sulfate binding in AAV2 [19] and is suitable for peptide display [10,12,13,14]. Similarly, AA442–469 generated another highly variable spike near the 3-fold axis in addition to the loop generated by AA561–588 (Figure 1B).

The training samples for our deep models are generated based on two public datasets described in previous work [7,18]. Ogden et al. [7] generated all single-codon mutants of the AAV2 cap gene and examined the effects of mutations systematically across all 735 positions. Their comprehensive codon-scanning approach provided sufficient information for the training of our models. Bryant et al. [18] generated multi-mutations at positions 561–588, which provided a multi-mutation training set for our model.

To verify our deep learning models, we generated test sequences following the scheme described in [7,18], which employs random mutations and rational design mutations on the wild-type AAV2 *Cap* sequence, including amino acid substitutions, insertions, or deletions involving at least 1 site and up to 5 sites at positions AA442–469. Since it is impractical to generate all possible variants exhaustively, we sampled about 50,000,000 variants from the entire search space, which was sufficient for the verification and potential valuable sequence discovery. These sequences were then embedded into a high-dimension space using an embedding matrix. At this stage, our goal was to predict whether the variants could be assembled to the level of the wild-type AAV2 capsid. We designed several transformer-based and convolutional neural network (CNN)-based models to predict the possibility of the successful assembly of the variants, and the results are ensembled (Figure 1C). Here, we have to emphasize that, in our multi-mutation sequence generation and experiments, our target positions were AA442–469—which were completely different from the AA561–588 described in previous works and have no overlap with the previous libraries. The highly dimensional data of the generated sequences were then fed into the models for prediction.

### 2.2. Construction of Plasmid Library Using Two-Step Cloning

**Figure 2:**
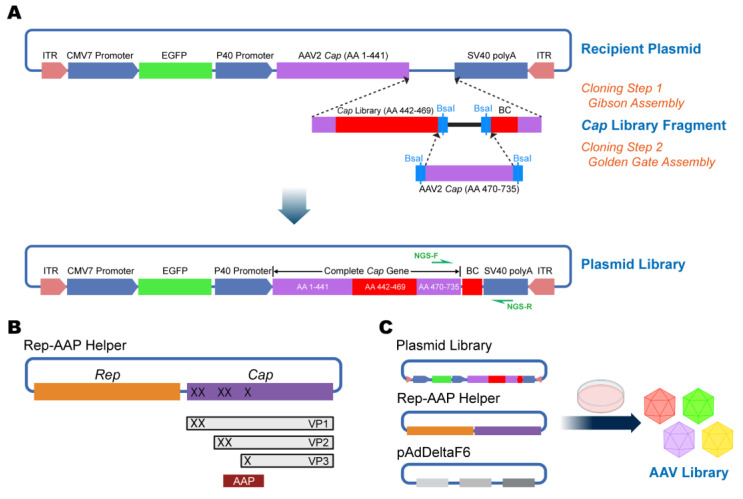
Construction of plasmid systems for AAV library preparation. (**A**) The plasmid library was constructed using two-step cloning. Step 1, *Cap* gene library fragments, generated by customized oligonucleotide synthesis, were inserted into the recipient backbone plasmid through Gibson assembly. Step 2, AA470–735 fragments of the AAV2 *Cap* gene were inserted into the backbone via Golden Gate assembly to generate a complete *Cap* reading frame. AA, amino acid. (**B**) The schematic shows the genome structure of the Rep-AAP helper plasmid. Stop codons were inserted into the *Cap* gene to terminate the expression of the VP1, VP2, and VP3 proteins, while the expression of the AAP protein was not affected. (**C**) An AAV mutant library was produced using the triple-transfection method in HEK293T cells.

*Cap* library fragments were synthesized on a customed oligo array (GENEWIZ, Suzhou, China), and each specific design sequence corresponded to a unique barcode (BC) for subsequent sequencing, with two BsaI restriction sites incorporated between the *Cap* library and the BC for Golden Gate assembly (Figure 2A). We pre-built the “recipient plasmid” as a backbone, which contained the 1–441 AA sequence of the AAV2 *Cap* gene. To complete the plasmid library construction, the first cloning step was to insert the synthesized *Cap* library fragment onto the recipient plasmid through Gibson assembly using the Gibson Assembly Master Mix (New England Biolabs, Ipswich, MA, USA, E2611). The second cloning step was to amplify the 470–735 AA fragments of the AAV2 *Cap* gene from pAAV2/2 (Addgene, Watertown, MA, USA, 104,963) and, then, to insert them into the backbone via Golden Gate assembly using Golden Gate Assembly Mix (New England Biolabs, Ipswich, MA, USA, E1601) to generate a complete *Cap* reading frame (Figure 2A). The primers NGS-F/R (Supplementary Table S1) were used to amplify the barcodes for the sequencing assay.

To provide the AAV2 Rep and assembly-activating protein (AAP), the construction of Rep-AAP helper was implemented on pAAV2/2 based on the design of Deverman et al. [11]. Five stop codons were introduced in the AAV2 *Cap* gene (at VP1 AAs: 6, 10, 142, 152, and 216) to eliminate VP1, VP2, and VP3 capsid protein expression; the stop codon at AA216 was designed to not disturb the codon in the alternative reading frame of the AAP protein (Figure 2B).

### 2.3. Cell Culture, AAV Preparation, and In Vitro Transduction Assay

HEK293T cells were purchased from the American Type Culture Collection (Manassas, VA, USA). Daily maintenance, cell preparation prior to viral packaging, and the triple-plasmid transfection of conventional viruses were completed as previously described [22].

For AAV mutant library production, the plasmid library, together with Rep-AAP helper and pAdDeltaF6 (Addgene, Watertown, MA, USA, 112,867), constituted a special triple-plasmid system (Figure 2C). For individual AAV packaging, the AAV2 plasmid (pAAV2/2) was obtained from Addgene (Watertown, MA, USA, 104,963), while mutant AAV plasmids were derived from the AAV2 plasmid using the Fast Mutagenesis System (TransGen Biotech, Beijing, China, FM111). The primer sequences (Mu-A1-F/R and Mu-A2-F/R) for site-directed mutagenesis, are shown in Supplementary Table S1. All viral vectors were aliquoted and stored at −80 °C until use.

For in vitro transduction assay, the cells were seeded for 16 h in 24-well plates at 70% confluence before transduction. Then, the medium was replaced by Dulbecco’s minimum essential media (DMEM) with 2% fetal bovine serum (FBS), and the AAVs were added into plates to infect these cells at a multiplicity of infection (MOI) of 35,000. At 24 h and 48 h post-infection, the EGFP signals were observed using an Olympus IX71 fluorescence microscope (Olympus, Tokyo, Japan).

### 2.4. Next-Generation Sequencing (NGS) Assay

The DNA specimens amplified with NGS-F/R primers (Supplementary Table S1) were utilized as input material for DNA sample preparation. Sequencing libraries were constructed using the NEB Next Ultra DNA Library Prep Kit for Illumina (New England Biolabs, Ipswich, MA, USA). In brief, the DNA specimens underwent end repair, A-tailing, and ligation with a full-length adapter for Illumina sequencing. Following the purification of the DNA products using the AMPure XP system (Beckman Coulter, Beverly, MA, USA), DNA concentration was determined using a Qubit 3.0 Fluorometer (Invitrogen, Carlsbad, CA, USA). The library size distribution was analyzed using an Agilent 2100 Bioanalyzer and quantified via real-time qPCR.

The index-coded specimens were clustered using a cBot Cluster Generation System, and then the library preparations were sequenced on an Illumina Novaseq 6000 platform (Novogene, Beijing, China), generating paired-end reads.

### 2.5. Research Animals and Stereotactic Intracerebral Injection

The 8–10-week-old C57BL/6 mice (Hunan SJA Laboratory Animal Company, Changsha, China; *n* = 3 in each group; *n* = 21 in total) were anesthetized using 1% pentobarbital intraperitoneally (i.p., 50 mg/kg of their body weight) and placed in a stereotaxic apparatus (RWD, Shenzhen, China). The injection coordinates referred to Paxinos and Franklin’s The Mouse Brain in Stereotaxic Coordinates, 4th edition [23]. The coordinate of the caudate putamen (CPu) was 0.75 mm anterior relative to bregma, 2.00 mm lateral, and 3.30 mm below the dura. The virus was injected at a rate of 0.03 μL/min using a stereotaxic injector equipped with a pulled glass capillary (Stoelting, Wood Dale, IL, USA, 53,311). After the injection was completed, the micropipette was held for an additional 10 min before being withdrawn. Animals were allowed to recover from anesthesia on a heating pad. For the intravenous administration, C57BL/6 mice were injected with AAV vectors at a dose of 4 × 10^11^ VG/mouse. Three weeks after injection, we sacrificed the mice to collect brain tissue via transcardiac perfusion with phosphate-buffered saline (PBS) and 4% paraformaldehyde.

### 2.6. Slice Preparation and Imaging

Brain slice preparation and imaging were completed according to the previously reported methods [22,24]. The antibodies used were rabbit anti-NeuN (neuronal nuclei antibody; 1:800; Abcam, Cambridge, MA, USA), goat anti-GFAP (glial fibrillary acidic protein antibody; 1:800; Abcam, Cambridge, MA, USA), Cy3-conjugated goat anti-rabbit immunoglobulin G (IgG) (1:400; The Jackson Laboratory, Bar Harbor, ME, USA) and rabbit anti-goat IgG conjugated with Cy3 (1:400; The Jackson Laboratory, Bar Harbor, ME, USA). The sections were incubated with the primary antibody overnight at 4 °C. After washing 3 times with PBS, the slices were incubated with the secondary antibody for 1 h at 37 °C. After washing with PBS, all the brain slices attached to the microscope slides were counterstained with 4′,6-diamidino-2-phenylindole (DAPI) (1:4000; Beyotime, Shanghai, China) and sealed with 70% glycerol. Imaging was performed using an Olympus VS120 virtual microscopy slide scanning system (Olympus, Tokyo, Japan) or a Leica TCS SP8 confocal microscope (Leica, Wetzlar, Germany).

### 2.7. Data Analysis and Protein Structure Prediction

GraphPad Prism 7.0 (GraphPad Software, La Jolla, CA, USA) was used for data analysis. One-sixth of the brain slices from each animal were selected for cell counting. The positive cells were quantified using ImageJ v1.8.0 (National Institutes of Health, Bethesda, MD, USA). All statistical data were presented as the mean ± the standard error of the mean (SEM). Statistical significance was set as *, *p* < 0.05; **, *p* < 0.01; ***, *p* < 0.001; and ****, *p* < 0.0001.

The amino acid sequence alignment was presented using Geneious Prime v2021.2 (Biomatters, Auckland, New Zealand). The VP3 protein structure predictions for AAV2.A1 and AAV2.A2 were achieved using ColabFold v1.5 (https://github.com/sokrypton/ColabFold, accessed on 31 January 2023) [25]. The three-dimensional structure alignment of the VP3 protein of AAV2, AAV2.A1, and AAV2.A2 was demonstrated using UCSF ChimeraX v1.4 [20,21] (UCSF, San Francisco, CA, USA).

## 3. Results

### 3.1. Design and Evaluation of Computer-Aided Directed Evolution

**Figure 3:**
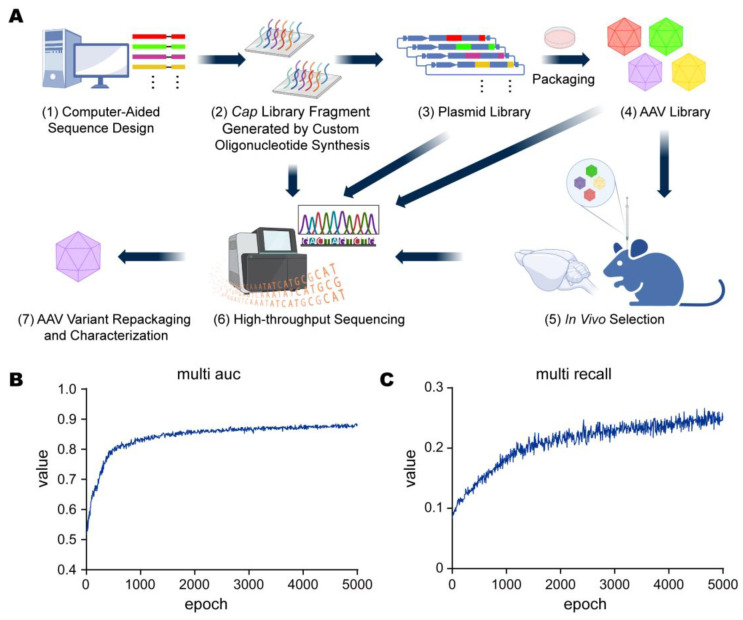
Paradigm design and evaluation of machine learning models. (**A**) Experimental workflow. (1) A *Cap* gene library of variants within AA442–469 was generated using computer-aided design. (2) The designed sequences (95,995) and the corresponding barcodes were synthesized in parallel on a custom oligonucleotide array. (3) A plasmid library was generated using two-step cloning. (4) The AAV library was manufactured through triple-transfection in HEK293T cells and characterized by high-throughput sequencing. (5) The AAV library was injected into C57BL/6 mice (*n* = 3) and, after 3 weeks of expression, tissues were harvested and the DNA was isolated, amplified, and prepared for sequencing. (6) After NGS, variants were ranked based on enrichment in select tissues. (7) Top enriched variants were repackaged and characterized systematically. (**B**) The AUC curve of the training of a single deep learning model. The AUC increased as the training proceeded. (**C**) The recall curve of the training. We empirically set the training batch size as 4096 and employed a step learning rate (LR) scheduler. The LR scheduler had an initial learning rate of 0.0001, the step size was set as 800, and gamma was set to 0.9.

We developed a systematic approach that combined computational design with in vivo selection to obtain novel AAV variants (Figure 3A). Our deep learning model showed promising results. The models were trained on both single-mutation (442–470) and multi-mutation (561–588) datasets, as mentioned in the public datasets. We then used the ensembled models to predict whether the mutated sequences could assemble, meaning that the virion assembly efficiency would reach the capsid assembly efficiency of the wild-type AAV2 capsid.

We generated 95,995 sequences that the models rated as could be assembled; among them, about 1/3 were generated with random multi-mutation, 1/3 were generated through rational design, and 1/3 were considered viable by our deep learning models. Based on the experiment, the average assembly rate was 22.57%, while random multi-mutation had an assembly rate of 7.20%, the rational design had an assembly rate of 11.70%, and AI-guided sequences had an assembly rate of 54.15%. This meant that nearly every 1 out of 2 sequences generated by models could actually be assembled, while the rate was less than 8% among the randomly mutated sequences. Compared to conventional methods, our approach greatly helped increase the efficiency of AAV mutant design, which conserves a large number of experiment resources. These results confirm that the computer-aided design strategy greatly improves the efficiency of library packaging, which is in line with previous reports [7].

For the training of deep learning models, we used the area under the curve (AUC) and recall rate analyses to evaluate the performance of the models (Figure 3B,C). We assume that recall rate is a well-known concept. AUC stands for the area under the receiver operating characteristic (ROC) curve and can be seen as a scale-invariant proxy of ROC. The ROC curve is a common metric used to evaluate the performance of a classification model. A ROC curve plots the true positive rate (TPR) vs. the false positive rate (FPR) at different classification thresholds. The AUC ranges in value from 0 to 1. As can be easily deduced, the higher the AUC is, the more accurate the model is.

**Figure 4:**
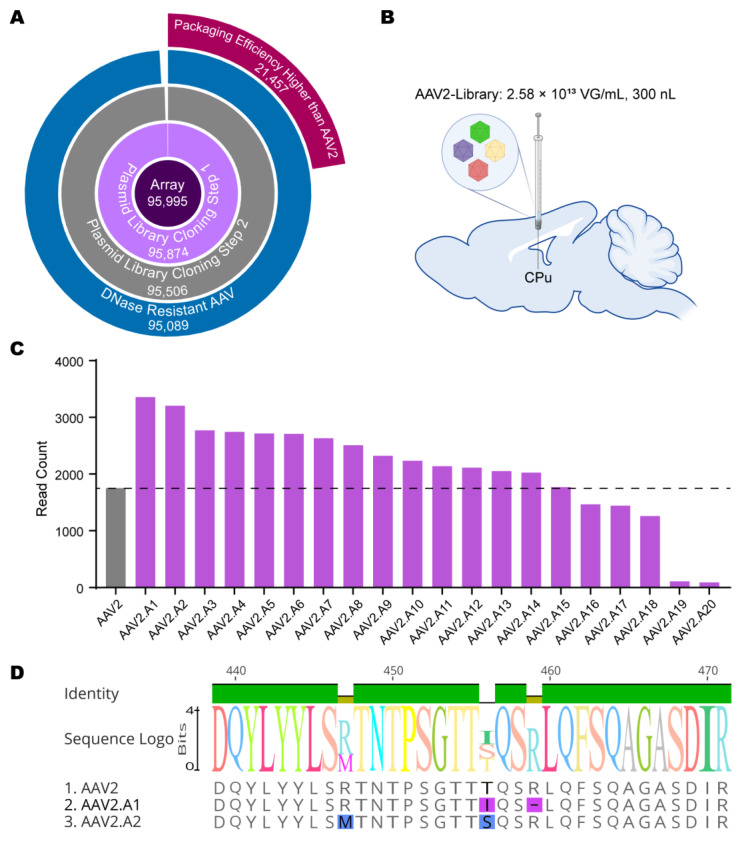
Diversity characterization during library preparation and mutant enrichment during in vivo screening. (**A**) Polar plot showing the absolute quantities of the unique mutant collected at each stage of the process. (**B**) In vivo screening for AAVs with high transduction efficiency; 7.74 × 10^9^ vector genomes (VG) of the AAV library were injected into the caudate putamen (CPu) of C57BL/6 mice (*n* = 3). (**C**) The top 20 enriched variants, detected using NGS during in vivo screening. (**D**) The amino acid sequence alignments for AAV2, AAV2.A1, and AAV2.A2.

Diversity at each step was detected using NGS. The *Cap* gene library sequences (95,995) and the corresponding barcode sequences (17 bp) were designed using a computer and synthesized in a custom oligonucleotide array. After two-step cloning, the plasmid library covered approximately 99.49% (95,506) of the design sequences. The viral library covered 99.06% (95,089) of the sequences and had 22.57% mutants (21,457) whose packaging efficiencies were higher than AAV2 (Figure 4A).

### 3.2. In Vivo Library Selections Converged on Dominant AAV Variants

The AAV library was injected into the caudate putamen (CPu) brain region of C57BL/6 mice (Figure 4B). After 3 weeks of expression, the tissues were harvested, and the DNA was isolated, amplified, and prepared for sequencing. Variants were ranked based on enrichment in the selected tissues. The top 20 variants identified via NGS sequencing were designated AAV2.A1 to AAV2.A20 based on their ranking—among which, 15 variants had in vivo transduction levels exceeding that of wide-type AAV2 transduction (Figure 4C). We further specifically characterized the top two mutants as AAV2.A1 and AAV2.A2. Compared to AAV2, AAV2.A1 had one amino acid substitution (T456I) and one amino acid deletion (R459Δ), and AAV2.A2 had two amino acid substitutions (R447M and T456S) (Figure 4D).

To visualize the 3D structural changes of the protein due to amino acid mutations, we used ColabFold v1.5 (https://github.com/sokrypton/ColabFold, accessed on 31 January 2023) [25] to predict the VP3 protein structure of AAV2.A1 and AAV2.A2. This was possible because the atomic structure of the AAV2 capsid was experimentally determined and the number of mutations in AAV2.A1 and AAV2.A2 was small. ColabFold, which incorporates homology modeling methods, was well-suited for this task. It should be noted that this software-based structural prediction method was intended to be a reference and not an in-depth investigation. The results showed that the two mutants had only minor structural changes compared to AAV2 (Supplementary Figure S1).

### 3.3. Novel AAV Variants Exhibited Higher Transduction Efficiency Than AAV2 In Vitro

**Figure 5:**
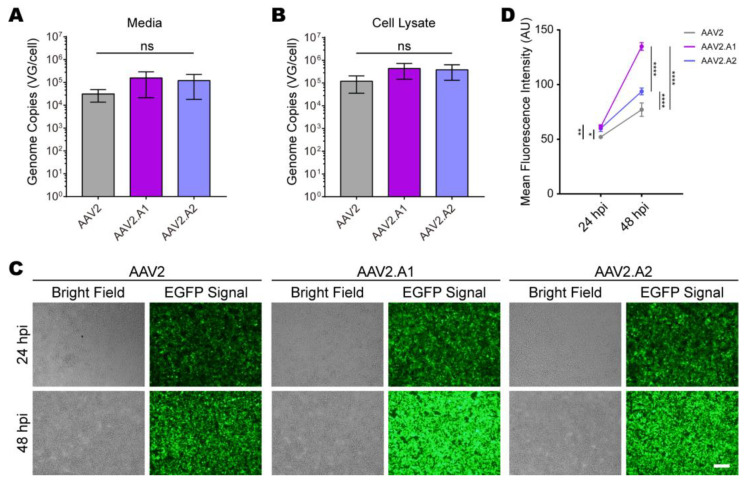
Characterization of the production and transduction properties of novel AAV variants. (**A**,**B**) Crude lysate PCR assays were performed on media (**A**) and cellular lysates (**B**) of HEK239T cells subjected to triple transfection for AAV2, AAV2.A1, and AAV2.A2. Values represent mean ± SEM, *n* = 3/group. Statistical analysis was completed using one-way ANOVA analysis followed by Tukey’s multiple comparisons test with an alpha value of 0.05. ns, no significant difference. (**C**) Fluorescence expressions of AAV2, AAV2.A1, and AAV2.A2 after infection into HEK293T cells for 24 h and 48 h, respectively. Scale bar = 200 μm. (**D**) Quantification of mean fluorescence intensities of different AAV transduction in vitro. Statistical analysis was completed using two-way ANOVA followed by Tukey’s multiple comparisons test with an alpha value of 0.05. AU, arbitrary units; hpi, hours post-infection; *, *p* < 0.05; **, *p* < 0.01; ****, *p* < 0.0001.

To systematically characterize AAV2.A1 and AAV2.A2, these two variants were individually repackaged using the traditional triple-plasmid transfection method, and their packaging efficiencies were evaluated. We found that the mutations of the amino acid sequences did not significantly affect the packaging efficiency of AAV2.A1 or AAV2.A2 compared to AAV2 (Figure 5A,B).

To evaluate the transduction efficiency of novel AAV variants in vitro, we measured the mean fluorescence intensity, expressed by AAV2-, AAV2.A1-, and AAV2.A2-infected HEK293T cells carrying the EGFP reporter driven by the CMV promoter. At 24 h post-infection, both variants showed higher fluorescence expression than AAV2. This difference became even more pronounced at 48 h post-infection, with AAV2.A1 being 2-fold higher than AAV2, in particular. (Figure 5C,D).

### 3.4. Novel AAV Variants Exhibited Higher Transduction Efficiency Than AAV2 In Vivo

**Figure 6:**
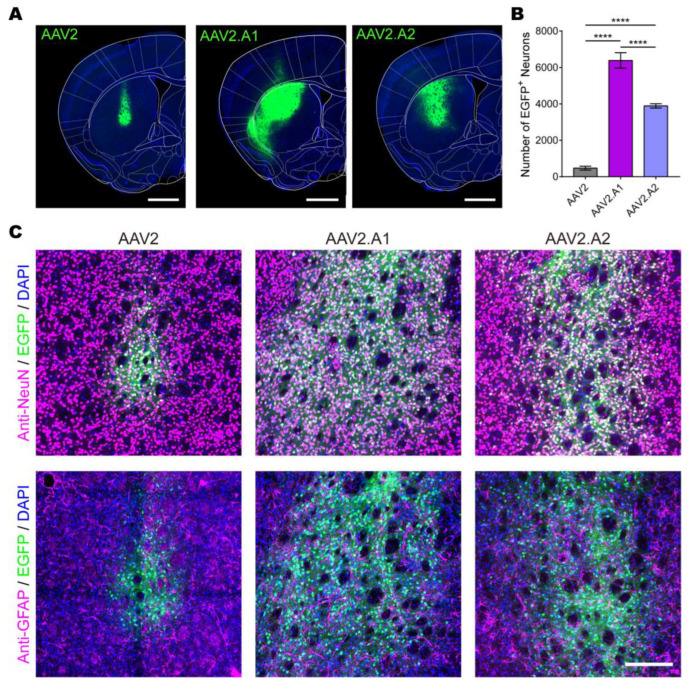
Characterization of transduction properties of novel AAV variants. (**A**) The fluorescence distribution of EGFP at the caudate putamen (CPu). AAV2-CMV-EGFP, AAV2.A1-CMV-EGFP, or AAV2.A2-CMV-EGFP viruses (300 nL, 3 × 10^9^ VG, in total) were injected into the CPu of C57BL/6 mice (*n* = 3 in each group, *n* = 9 in total). Scale bar = 1 mm. (**B**) Quantitative analysis of the number of EGFP^+^ cells transduced with different AAV vectors in CPu. Statistical analyses were completed using one-way ANOVAs followed by Tukey’s multiple comparisons tests with an alpha value of 0.05. ****, *p* < 0.0001. (**C**) Immunofluorescence of cell types by neuronal nuclei antibody (anti-NeuN) or glial fibrillary acidic protein antibody (anti-GFAP). DAPI, 4′,6-diamidino-2-phenylindole. Scale bar = 200 μm.

To assess the in vivo transduction efficiency of the AAV variants, we injected AAV2.A1 and AAV2.A2 carrying an EGFP reporter controlled by the CMV promoter into the CPu for comparison with AAV2. Both AAV2.A1 and AAV2.A2 showed larger transduction ranges than AAV2, with 15 and 10 times more EGFP-positive neurons in the CPu region, respectively (Figure 6A,B). We also tested the ability of AAV2.A1 and AAV2.A2 to cross the blood–brain barrier via tail vein injections in adult C57BL/6 mice (*n* = 3 in each group); no fluorescent signals were observed in the mouse brain slices (Supplementary Figure S2).

Then, the GFAP and NeuN antibodies were used to identify cell types via immunofluorescence. Our results demonstrated that the vast majority of the EGFP-positive cells were neurons, and astrocytes were almost absent for AAV2, AAV2.A1, and AAV2.A2 (Figure 6C). Since the vast majority of the cells that AAV2 infects in the CNS are neurons [26], limited amino acid mutations in the variants did not alter the cell-targeting specificity of AAV2.

## 4. Discussion

We established a computer-aided directed evolution system paradigm and designed a gene mutation library through machine learning. Then, we used two-step cloning to complete the construction of a plasmid library and packaged a high-diversity AAV mutation library. As proof of principle, two AAV2 variants—named AAV2.A1 and AAV2.A2—with higher transduction efficiencies than AAV2, were obtained after in vivo screening.

The targeted mutagenesis of the variable regions (VRs) exposed to the capsid surface is a common sense approach, as these regions are the least evolutionarily conserved sequences and are known to mediate virus–receptor interactions [27]. The prominence of capsid protrusions is also utilized in the most rational way, taking advantage of the flexibility of its surface-exposed loops, especially VR-IV and VR-VIII [27]. A large number of studies have been conducted to construct mutant libraries, develop rational designs by inserting short peptides into the AAV capsid VR-VIII, and obtain AAV variants with excellent characteristics, such as AAV2-7m8 [10], AAV2-retro [13], AAV-MNM004 [14], AAV-PHP.B/AAV-PHP.eB [11,28], MyoAAV [29], AAV9-Retro [30], and AAV-ie [31]; however, only a few studies of AAV capsid engineering have involved the VR-IV [32,33]. Based on the data previously collected by the George Church group [7,18], our study constructed a mutation library in the vicinity of VR-IV through computer-aided design and obtained novel variants with high transduction efficiency through in vivo selection, thereby confirming the research potential of AAV vector engineering in this region.

During the process of library construction, the paradigm of computer-aided directed evolution designed in this study has shown significant advantages. Our deep learning models showed prominent performance in predicting the viability of the random multi-mutated sequences, which greatly conserves experimental resources, and thus, removes almost all restrictions in sequence generation since we can confidently focus on the high-scoring sequences without wasting significant effort on non-viable ones. That is, the loss of library diversity at each step was low, which greatly ensured the richness of the library (Figure 4A). Finally, an AAV library containing about 95,089 mutants was obtained, which laid the foundation for subsequently more refined library screening.

As proof of principle, we first screened and then characterized two variants from the library, AAV2.A1 and AAV2.A2, whose transduction efficiencies in the brain were 10~15 times higher than that of AAV2. This confirms the transformation potential of the AA442–469 region. Through further iterative evolution, we may obtain better variants. On the other hand, this also makes AAV2.A1 and AAV2.A2 new parents in engineering modification, and many variants obtained through peptide display on AAV2 could be transplanted into AAV2.A1 or AAV2.A2 to get better transduction effects.

Structural characterization showed that two amino acid changes in AAV2.A1 or AAV2.A2 did not significantly affect the three-dimensional structure (Supplementary Figure S2), and these sites have not been reported to directly bind to cell surface receptors in previous studies. The Ubiquitin–proteasome pathway plays a significant role in the intracellular trafficking of AAV, and the phosphorylation of certain surface-exposed amino acid residues on the capsid provides the primary signal for ubiquitination. Previously, Aslanidi et al. [34] improved the transduction efficiency of AAV2 through site-directed mutagenesis replacing AAV2 surface-exposed threonine (T) residues, including T455. Zhang et al. [35] reported that the AAV2 capsid undergoes a conformational shift after AAVR binding, and this movement forms close contact between AAVR F156 and AAV2 T456, which helps stabilize the virus–receptor interaction. Given that the substitution of T456 occurs in both AAV2.A1 and AAV2.A2, perhaps the modification of T456 affects the above process and changes the transduction effect of AAV2. For AAV2.A1, another mutation site is the deletion of R459. The mutation of AAV2 residue R459 was previously reported to increase cellular transduction three-fold [36]. In the combination of AAV2 and AAVR, R459 of AAV2 will lead to unfavorable steric and/or electrostatic interactions with K399 in the AAVR PKD1 domain [37], so the deletion of R459 may facilitate a better interaction of AAV2 and AAVR. For AAV2.A2, it is probably worth mentioning that R447 forms a salt bridge with E499 of VR-V of a symmetry-equivalent subunit [38], and residues 499–503 contact the receptor AAVR [37]. This salt bridge is not conserved among all AAVR-binding serotypes; thus, it is fairer to note that R447 interacts with a region of AAV that is in direct contact. Based on the above information, a more detailed mechanism for the wider spread of AAV2.A1 and AAV2.A2 still depends on further exploration.

## Data Availability

All data are available upon reasonable request.

## Supplementary Materials

The following supporting information can be downloaded at: https://www.mdpi.com/article/10.3390/v15040848/s1. Table S1: Primer sequences used in the experiment; Figure S1: VP3 protein structural comparison of AAV2, AAV2.A1 and AAV2.A2. Structural superposition of AAV2 (light grey), AAV2.A1 (light purple) and AAV2.A2 (light blue) shown as ribbon diagrams. The VP3 protein structures of AAV2.A1 and AAV2.A2 were predicted via ColabFold v1.5 (https://github.com/sokrypton/ColabFold, accessed on 31 January 2023) [25], enlarged view shows the structural changes by the mutants T456I and R459Δ in the AAV2.A1 capsid and the mutants R447M and T456S in the AAV2.A2 capsid. This figure was generated using UCSF ChimeraX v1.4 [20,21] (UCSF, San Francisco, CA, USA); Figure S2: Variants obtained from library screening did not exhibit cross-blood-brain barrier effects. EGFP-expressing AAV2, AAV2.A1 or AAV2.A2 (5 × 10^11^ VG/mouse) was intravenously injected into adult C57BL/6 mice (n = 3 in each group, n = 9 in total). Scale bar = 1 mm.
