# Supplementary material for: Computer-Aided Directed Evolution Generates Novel AAV Variants with High Transduction Efficiency
Source: Viruses. 2023 Mar 26;15(4):848. doi: 10.3390/v15040848 (PMC10143561; doi:10.3390/v15040848)
Supplement: Supplementary file 1 [file viruses-15-00848-s001.zip › viruses-2221499-supplementary.pdf]

Supplementary Information

(Contains Supplementary Table S1 and Supplementary Figures S1-S2 with Legends)

Supplementary Table S1. Primer sequences used in the experiment

No.	Primer Name	Sequence
1	NGS-F	CCAACTACAACAAGTCTGTTAATGTGGACT
2	NGS-R	ATCTTACACATCTCCCCCTGACATATG
3	Mu-A1-F	AAGTGACTGGATAGTGGTTCCACTTGGA
4	Mu-A1-R	ACTATCCAGTCACTTCAGTTTTCTCAGGC
5	Mu-A2-F	ATGACAAACACTCCAAGTGGAACCACCTCCCAGTCAAGGCTTCA
6	Mu-A2-R	GAGGTGGTTCCACTTGGAGTGTTTGTCATGCTCAAGTAATACAGGT

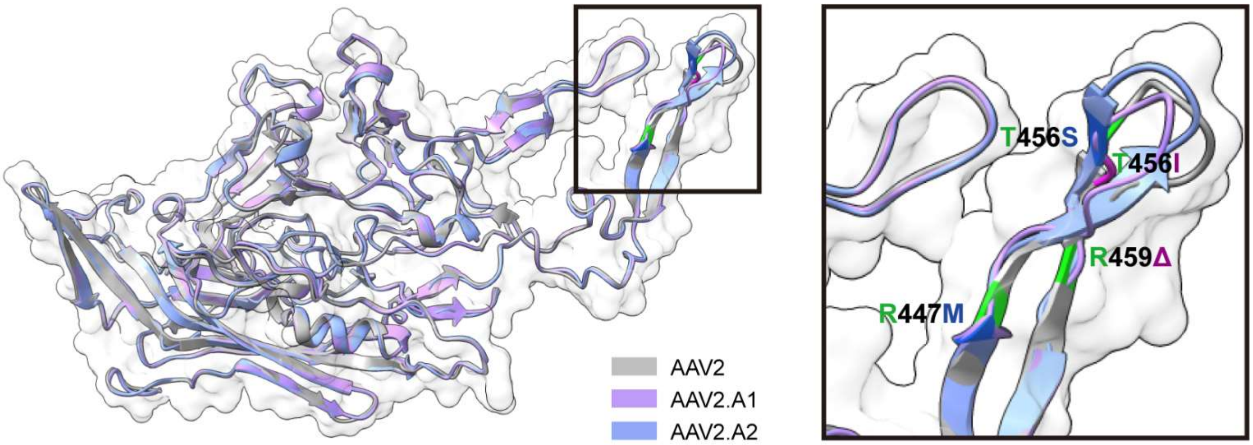

Supplementary Figure S1. VP3 protein structural comparison of AAV2, AAV2.A1 and AAV2.A2. Structural superposition of AAV2 (light grey), AAV2.A1 (light purple) and AAV2.A2 (light blue) shown as ribbon diagrams. The VP3 protein structures of AAV2.A1 and AAV2.A2 were predicted via ColabFold v1.5 (https://github.com/sokrypton/ColabFold) [25], enlarged view shows the structural changes by the mutants T456I and R459Δ in the AAV2.A1 capsid and the mutants R447M and T456S in the AAV2.A2 capsid. This figure was generated using UCSF ChimeraX v1.4 [20,21] (UCSF, San Fran-cisco, CA, USA).

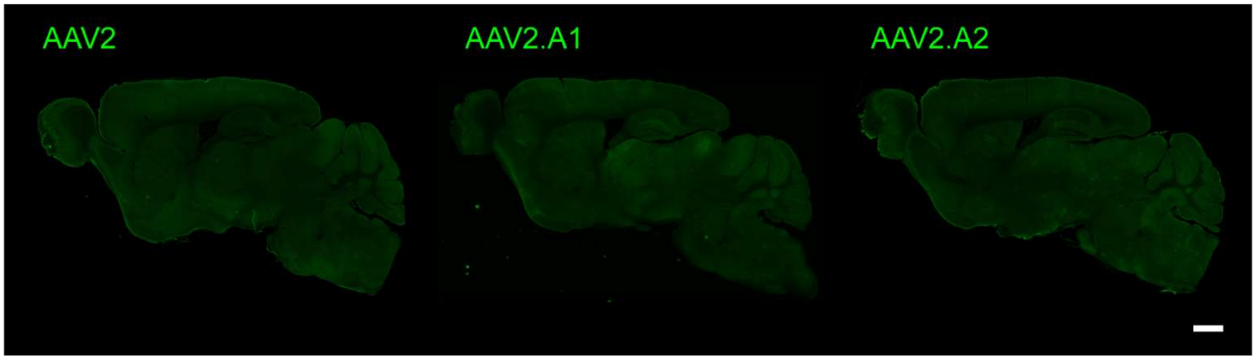

Supplementary Figure S2. Variants obtained from library screening did not exhibit cross-blood-brain barrier effects. EGFP-expressing AAV2, AAV2.A1 or AAV2.A2 (5×10^{11} VG/mouse) was intravenously injected into adult C57BL/6 mice (n = 3 in each group, n = 9 in total). Scale bar = 1 mm.
